# Is the EuroSCORE II reliable to estimate operative mortality among octogenarians?

**DOI:** 10.1371/journal.pone.0187056

**Published:** 2017-11-16

**Authors:** Sophie Provenchère, Arnaud Chevalier, Walid Ghodbane, Claire Bouleti, Philippe Montravers, Dan Longrois, Bernard Iung

**Affiliations:** 1 Département d’Anesthésie-Réanimation, APHP, Hôpital Bichat-Claude Bernard, Paris, France; 2 INSERM Centre d’Investigation Clinique 1425, APHP, Hôpital Bichat-Claude Bernard, Paris, France; 3 Département de Chirurgie Cardiaque, APHP, Hôpital Bichat-Claude Bernard, Paris, France; 4 Département de Cardiologie, APHP, Hôpital Bichat-Claude Bernard, Paris, France; 5 Université Paris 7-Diderot, Paris, France; 6 INSERM 1148, Paris, France; University of Padova, ITALY

## Abstract

**Objectives:**

Concerns have been raised about the predictive performance (PP) of the EuroSCORE I (ES I) to estimate operative mortality (OM) of patients aged ≥80. The EuroSCORE II (ES II) has been described to have better PP of OM but external validations are scarce. Furthermore, the PP of ES II has not been investigated among the octogenarians. The goal of the study was to compare the PP of ES II and ES I among the overall population and patients ≥ 80.

**Methods:**

The ES I and ES II were computed for 7161 consecutive patients who underwent major cardiac surgery in a 7-year period. Discrimination was assessed by using the c- index and calibration with the Hosmer-Lemeshow (HL) and calibration plot by comparing predicted and observed mortality.

**Results:**

From the global cohort of 7161 patients, 832 (12%) were ≥80. The mean values of ES I and ES II were 7.4±9.4 and 5.2±9.1 respectively for the whole cohort, 6.3±8.6 and 4.7±8.5 for the patients <80, 15.1±11.8 and 8.5±11.0 for the patients ≥80. The mortality was 9.38% (≥80) versus 5.18% (<80). The discriminatory power was good for the two algorithms among the whole population and the <80 but less satisfying among the ≥80 (AUC 0.64 [0.58–0.71] for ES I and 0.67 [0.60–0.73] for the ES II without significant differences (p = 0.35) between the two scores. For the octogenarians, the ES II had a fair calibration until 10%-predicted values and over-predicted beyond.

**Conclusions:**

The ES II has a better PP than the ES I among patients <80. Its discrimination and calibration are less satisfying in patients ≥80, showing an overestimation in the elderly at very high-surgical risk. Nevertheless, it shows an acceptable calibration until 10%- predicted mortality.

## Introduction

The remarkable gain in life expectancy in most developed countries is challenging for the medical community and for public health decision makers. For both men and women, life expectancy is steadily increasing and the population aged 80 years and over is expected to more than triple in 2060 [1]. Despite disparities among countries, the increased percentage of elderly individuals is largely due to a decrease in cardiovascular disease mortality in high income countries [2]. Consequently, the number of interventions performed in older patients is increasing. Moreover, in this specific population the presence of multiple comorbidities and chronic multiple drugs therapy make preoperative risk assessment crucial, though complex [1, 3].

In cardiac surgery, the number of octogenarians has increased in the past 20 years and now represents more than 10 per cent of the cardiac surgical patients in developed countries. In this specific population at higher risk of major morbidity or mortality [4], risk stratification is even more important since credible alternatives to conventional surgery such as transcatheter aortic valve implantation (TAVI) or percutaneous mitral edge-to-edge repair (MitraClip^®^) exist.

Patient selection is nowadays based on both individual clinical judgment and the Heart Team, a standard of care [5], which integrates current scoring systems, the original EuroSCORE [6] (ES I) and Society of Thoracic Surgeons Predicted Risk of Mortality (STS PROM). It has been demonstrated that the ES I [6] tended to overestimate mortality among high-risk cardiac surgery patients [7]. Consequently, a new scoring system, the EuroSCORE II (ES II), has been developed in order to achieve a better calibration [8]. Numerous studies of external validation have demonstrated that ES II achieved a good discrimination and an improved calibration except among patients with a predicted OM of more than 30 per cent [9, 10]. However, few studies have focused specifically on the predictive performance (PP) of the ES II among high risk patients and in the elderly.

The objective of this study was therefore to evaluate the discrimination and the calibration of the ES II in a contemporary cardiac surgical population and to compare its performance with the ES I among the overall population and the elderly, defined as ≥ 80 years old.

## Material and methods

### Study population and study design

The study population included all consecutive adult patients who underwent cardiac surgery with cardiopulmonary bypass (CPB) over a 7-year period between January 2006 and December 2012 (7161 patients enrolled) in our institution. Since 2006, patients, surgery characteristics as well as in-hospital outcomes were prospectively entered into an institutional database by trained research assistants (S1 Table).

The study was approved by Bichat hospital Institutional Review Board (“Comité d’Evaluation des Projets de Recherche Biomédicale Paris Nord”; IRB 00006477). It waived the need for patient’s informed consent because the data were analyzed anonymously. In accordance with the French Law, the registry was approved by the Advisory Committee for Information Processing in Health Research (Comité Consultatif sur le Traitement de l'Information en matière de Recherche dans le domaine de la Santé, Paris, France) and the French National Commission on Computing and Liberty (Commission Nationale Informatique et Liberté, Paris, France). Reporting of the study complies with the Strengthening the Reporting of Observational studies in Epidemiology recommendations statement for reporting. Although data were prospectively acquired for a research purpose, the study should be considered as a retrospective study.

Analyses were performed for all types of surgical procedures including emergency. The types of surgery were defined as follows: isolated coronary artery bypass (CABG), isolated valvular surgery or valvular surgery combined with CABG and other procedures.

The only exclusion criteria were patients less than 18 years old and cardiac transplantations.

The primary endpoint was in-hospital mortality. In-hospital mortality was defined as death occurring at any time in the hospital where the surgery was performed before discharge from hospital. The 30 days mortality was also recorded.

We analyzed separately the two subgroups of patients aged <80 and ≥80 years.

Medical records of non survivors among the octogenarians were retrospectively assessed by two senior cardiac anesthesiologists to determine the causes of death. Causes of death were categorized in 3 groups according to timing, i.e. Group 1: Early complications (D0-D2), Group 2: Acute complications (D3-D15) and Group 3: Delayed complications (≥D16). Definitions are given in Table 1.

**Table 1:** Causes of death and time to death.

Causes of death
**Group 1: Early complications (D0-D2)**
Deceased in the operating room Severe and uncontrolled bleeding with massive transfusion
**Group 2: Acute complications (D3-D15)**
Cardiogenic shock (e.g., multiple high dose inotropes, mechanical cardio-circulatory support) Hemorragic shock Sepsis or septic shock Massive stroke or mesenteric ischemia Tamponnade Sudden death
**Group 3: Delayed complications (≥D16)**
Mediastinitis Endocarditis MOF Prolonged mechanical ventilation Withdrawal of care

The ES I and the ES II values were calculated for each patient according to patient characteristics using published equations [6, 8].

### Statistical analysis

Results are expressed as mean ± SD, number (percentage). Comparisons of continuous variables used unpaired Wilcoxon tests and comparisons of discrete variables Chi-2 or Fisher exact tests. The performance of the ES models was analyzed focusing on discrimination and calibration.

The discrimination performance indicates the extent to which the model distinguishes between patients who will die or survive. It was evaluated by constructing receiver operating characteristic curves for each model and calculating the area under the curve (AUC) with 95% confidence intervals (CI). Numerically, an area of 1.0 indicates the perfect discrimination power, whereas an area of 0.5 indicates no discrimination of the binary outcome.

Calibration refers to the agreement between observed and predicted in-hospital mortality. Overall model calibration was assessed by comparing observed and predicted mortality in 10 equally sized subgroups in increasing order of patient risk, according to the HL test for goodness of fit. A HL p values >0.05 indicates a well-calibrated model for the study population. Model calibration was also assessed using the observed/expected (O/E) mortality ratio. The 95% CI of O/E ratio was calculated using the Byar's method. An O/E ratio >1.0 indicates that the score underpredicts mortality and an O/E ratio <1.0 indicates that the score overpredicts mortality. The O/E ratio indicates good calibration if its 95% CI includes the value 1.0. Finally, model calibration was assessed visually according to the risk level by comparing predicted and observed mortality rates with 95% CI using calibration plots. Whereas the perfect calibrated prediction stays on the 45-degree line, a curve below or above the diagonal reflects overestimation and underestimation respectively. The P-value for statistical significance was 0.05. The statistical analyses were performed with SAS version 9.2 and SPSS version 19.0.

## Results

### General demographics

A total of 7161 consecutive patients were included in the study.

Table 2 summarizes patient profile with demographics, co morbidities and procedures performed as defined by EuroSCORE algorithms among the entire cohort and for both study groups.

**Table 2:** Characteristics for the whole population and for both age groups.

Patients characteristics	Overall population N(%)	Patients <80 N(%)	Patients ≥ 80 N(%)	*p-value*
		6329 (88)	832 (12)	
**Age (year)**	63±14 [18–94]	61±13 [18–79]	83±3 [80–94]	<0.0001
**Male gender**	4869 (68)	4447 (70)	422 (51)	<0.0001
**Body mass index (Kg/m**^**2**^**)**	26.5±4.6 [12–50.2]	26.5±4.6 [12–50]	26.1±4.25 [14.4–47.3]	0.0157
**Medical and surgical history**				
**Smoker**				
Current	1116 (15.6)	1099(17.3)	17 (2)	<0.001
Past	2534 (37.4)	2267 (35.8)	267(32)	
Unknown	30 (0.4)	24 (0.38)	6 (0,7)	
No	3481(48.6)	2939 (46.4)	542 (65)	
**Arterial hypertension**	4025 (56.2)	3416 (53)	609 (73)	<0.0001
**Diabetes status**				
Oral therapy only	1247(17.4)	1125 (18)	122 (14)	0.001
Insulin	565 (7.9)	519 (8)	46 (6)	
No	5349 (74.7)	4685 (74)	664 (80)	
**Dyslipemia**	3591 (50)	3145 (50)	445 (53)	0.039
**History of coronary artery disease**				
Stable angina	1279 (17.9)	1148 (18)	131 (16)	0.0002
STEMI	722 (10)	666 (10)	56 (7)	
Non STEMI	735 (10.3)	658 (10)	56 (7)	
No	4425 (61.8)	3857 (61)	568 (68)	
**ACS <90 days**	994 (13.8)	901 (14)	93 (11)	0.016
**Previous cardiac surgery**				
Others	53 0.7)	49 (0,8)	4 (0,5)	<0.0001
CABG	68 (0.9)	59 (1)	9 (1)	
Combined surgery	30 (0.4)	27 (0.4)	3 (0.3)	
Valvular surgery	574 (8)	550 (9)	24 (3)	
No	6436 (89)	5644 (89)	792 (95)	
**Extracardiac arteriopathy**				
Lower limbs	337 (4.7)	297 (5)	40 (5)	<0.0001
Previous vascular surgery	264 (3.7)	228 (4)	36 (4)	
Carotid stenosis >50%	382 (5.3)	308 (5)	74 (9)	
No	6178 (86.3)	5496 (86)	682 (82)	
**Chronic pulmonary disease**				
Untreated	282 (3.9)	240 (4)	42 (5)	<0.0001
Treated	413 (5.8)	337 (5)	76 (9)	
No	6466 (90.3)	5752 (91)	714 (86)	
**On dialysis**	71 (1)	70 (1)	1 (0,1)	0.0025
**Cancer**				
<5years	209 (2.9)	169 (3)	40 (5)	<0.0001
>5 years	260 (3.6)	210 (3)	50 (6)	
Metatstasis	20 (0.3)	16 (0.2)	4 (0.5)	
No Metatstasis	76 (0.1)	60 (0.11)	16 (0.2)	
**Atrial fibrillation**	850 (11.9)	718 (11)	132 (16)	0.0002
**Neurological or musculoskeletal dysfunction**^a^	212 (3)	193 (3)	19 (2)	0.270
**Previous stroke**				
TIA	199 (2.8)	167 (2)	32 (4)	0.1318
Hemorrhagic stroke	62 (0.9)	54 (1)	8 (1)	
Ischemic stroke	341 (4.8)	309 (5)	32 (4)	
No	6559 (91.6)	5799 (92)	760 (91)	
**Cirrhosis**				
Uncomplicated	30 (0.4)	30 (0.4)	0	0.044
PHT	45 (0.6)	43 (0.68)	2 (0.2)	
No	7086 (99)	6256 (99)	830 (99)	
**Active endocarditis**	321 (4.5)	299 (4.7)	22 (2.6)	0.006
**NYHA class**				
I	1538 (21.5)	1449 (23)	89(10)	<0.0001
II	672 (9.4)	639 (10)	33 (4)	
III	2789 (38.9)	2453 (39)	336 (40)	
IV	2162 (30.2)	1788 (28)	374 (45)	
**SPP >55 mmHg**	333 (4.7)	288 (5)	45 (5)	0.269
**SPP (mmHg)**	42± 14 [10–125]	41±14 [10–125]	44±12 [20–90]	0.269
**Critical preoperative state**^b^	218 (3)	198 (3)	20 (2)	0.253
**Type of procedure**				
CABG	2648 (37)	2485 (39)	163 (20)	<0.0001
Valvular surgery	4134 (57.7)	3490 (55)	645 (78)	
Other	378 (5.3)	353 (6)	24 (2)	
**Urgency**^c^	409 (5.7)	353 (6)	56 (7)	0.1778
**Creatinine clearance (ml/min/m**^**2**^**SC)**^d^	77.2±32 [5.5–262]	81.1±31 [5.5–262]	47±16 [6–108]	<0.0001
**LVEF (%)**	57.8±12 [10–89]	57.9±12 [10–89]	57.6±12 [15–84]	0.5313

***Abbreviations*: MI**: Myocardial Infarction; **TIA**: Transient Ischemic Attack; **STEMI**: ST elevation Myocardial Infarction; **ACS**: Acute Coronary Syndrome; **CABG**: Coronary Artery Bypass Grafting; **PHT**: Portal HyperTension; **NYHA**: New York Heart Association; **SPP**: Systolic Pulmonary Pressure; **BMI**: Body Mass Index; **LVEF**: Left Ventricular Ejection Fraction

^**a,b,c,d**^**°**: as defined by EuroSCORE algorithms

Continuous variables are presented as means ±SD and [minimum-maximum], categorial data are presented as number (percentage) of patients. P-values are from Wilcoxon tests and Chi-2 or Fisher exact tests.

Body mass index (BMI) was calculated using the formula:

$$\text{BMI}=\text{weight}\left(\text{kg}\right)/{\text{height}}^{2}({\text{m}}^{2}).$$

Creatinine Clearance as an estimate of Glomerular Filtration Rate (eGFR) was calculated using the Cockcroft–Gault formula:

eGFR(ml/min)=(140-age(years))×weight(kg)×0.85(iffemale)/72×serumcreatinine(mg/dL)

eGFR was standardized for 1.73 m^2^ of body surface area.

All the variables associated with mortality in the study of Nashef et al.[8][age, sex, extracardiac arteriopathy, chronic lung disease, poor mobility, previous cardiac surgery, creatinine clearance, active endocarditis, critical preoperative state, left ventricular function, systolic pulmonary artery pressure, urgency and type of procedure] are included in Table 2.

Results are given with the item “creatinine clearance” as proposed by the on-line EuroSCORE interactive calculator.

The elderly group (age≥80) embodying 832 patients had a higher prevalence of arterial hypertension, worse NYHA functional class and more frequent atrial fibrillation than those aged <80. They also had more previous chronic conditions such as malignancy, extra-cardiac arteriopathy, chronic pulmonary disease and previous stroke. Moreover, the only biological variable needed for ES II calculation, the creatinine clearance strongly differed between the two groups emphasizing the high prevalence of chronic kidney disease among the elderly. Finally, they differed from the younger patients by undergoing more frequently valvular surgery.

### ES and mortality

Our in-hospital mortality rate was 5.7%, 5.2% and 9.4% in the overall population, in patients aged <80 and ≥ 80 respectively.

The distribution of in-hospital mortality of the elderly according to the 3 groups of timing of deaths as defined in Table 1 was 25.6% (N = 20) in group 1 (mean ES II: 17.6±22.3), 38.4% (N = 30) in group 2 (mean ES II: 11.3±13.9) and 35.8% (N = 28) in group 3 (mean ES II: 24.1±28.2). Twelve patients (60%) in group 1 died in the operating room and 4 (15%) in group 3 died after withdrawal of care.

Table 3 reports observed mortality and ES values of our patient cohort and according to age subgroups:

For the whole cohort and the two subgroups (<80 and ≥ 80 respectively), there was a difference of nearly 1% between 30 day mortality and in-hospital mortality.
The mean values of ES I and ES II were 7.4±9.4 and 5.2±9.1 respectively for the whole cohort, 6.3±8.6 and 4.7±8.5 for the patients aged <80, 15.1±11.8 and 8.5±11.0 for the patients aged ≥80.
The ES I tended to overestimate in-hospital mortality among the whole cohort and for both study groups. There was in particular a strong overestimation of in-hospital mortality among the elderly.
Conversely, the predicted mortality of the ES II was close to the observed in-hospital mortality, even in the elderly.

**Table 3:** Observed and predicted mortality of the study population.

	Overall population	<80	≥80
**N** =	7161	6329	832
**In-hospital mortality**	406 (5.67)	328 (5.18)	78 (9.38)
**30 days mortality**	327 (4.57)	265 (4.18)	62 (7.45)
**EuroSCORE I**	7.36±9.43 [0.88–88.48]	6.35±8.57 [0.88–88.48]	15.06±11.85 [3.44–85.88]
**EuroSCORE II**	5.17±9.07 [0.49–94.42]	4.73±8.51 [0.49–94.42]	8.51±10.98 [1.19–89.31]

Continuous variables are presented as means ±SD and [95% CI]; categorial data are presented as percentage of patients.

### Performance of EuroSCORE

#### Discrimination

**Fig 1:**
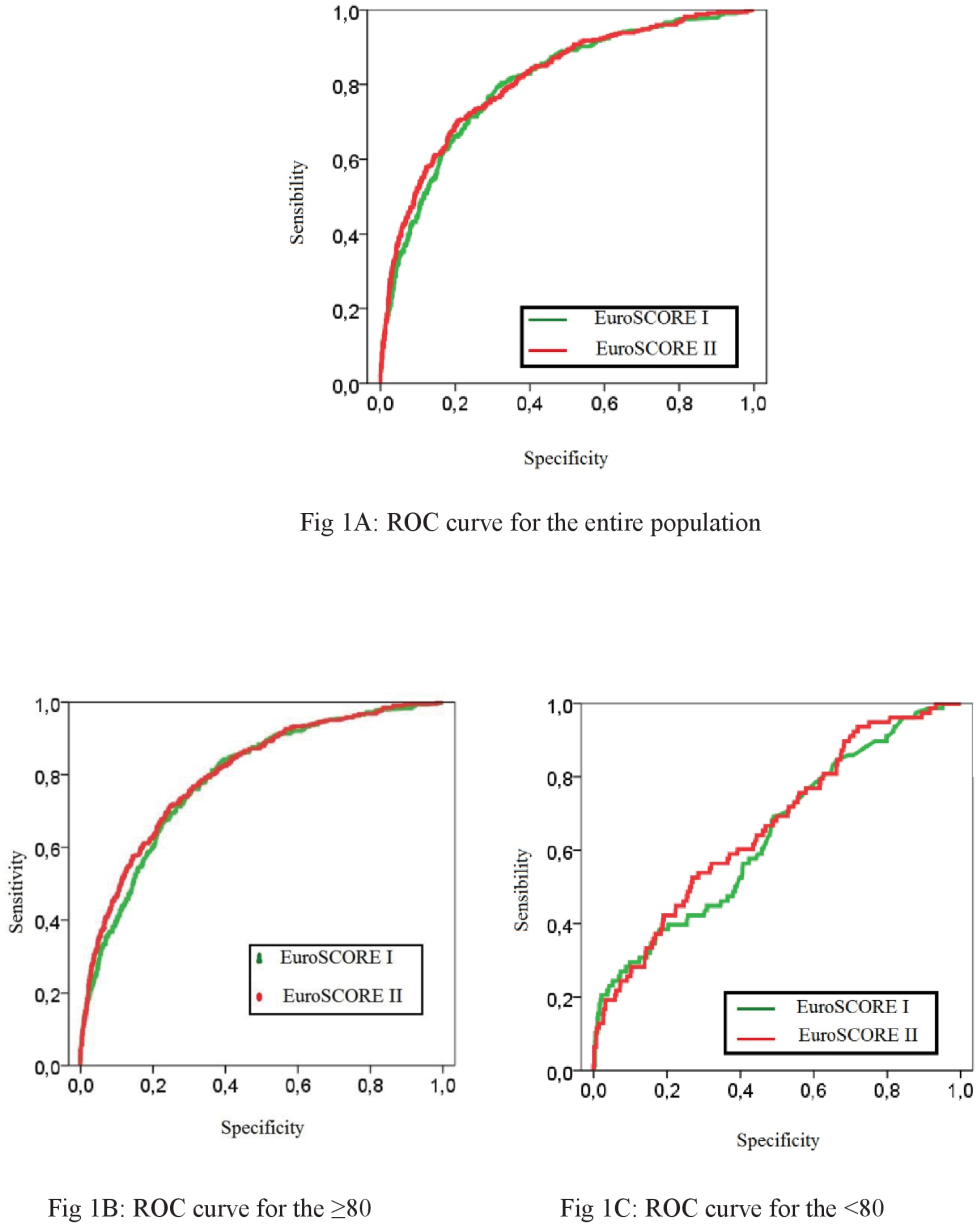
Receiver operating characteristics curves for the two models, for the entire population (Fig 1A) and for the two studied groups (Fig 1B: ≥80 and Fig 1C: <80).

The ROC curves for ES I and ES II are plotted in Fig 1.

The AUC was high in the overall population and among patients aged <80, being 0.79 [0.77–0.81] and 0.80 [0.78–0.83] for the ES I, respectively. When considering the ES II, the AUC was 0.80 [0.78–0.82] and 0.81 [0.79–0.84], respectively. The comparison among the two ES highlighted a trend to a better discrimination for ES II and the previous original version for the overall population (p = 0.07) but there was no significant differences for the patients aged <80 (p = 0.21).

Conversely, the discrimination was less satisfying for patients aged ≥80, being 0.64 [0.58–0.71] for ES I and 0.67 [0.60–0.73] for the ES II without significant differences (p = 0.35) between the two scores (Table 4).

**Table 4:** Discrimination for in-hospital mortality among the overall population and according to age.

	Overall population	<80	≥80
N =	7161	6329	832
c-index ES I	0.79 [0.77–0.81]	0.80 [0.78–0.83]	0.64 [0.58–0.71]
c-index ES II	0.80 [0.78–0.82]	0.81 [0.79–0.84]	0.67 [0.60–0.73]
p ES I/ES II	0.07	0.21	0.35

Continuous variables are presented as means ±SD and [95% CI]; P-values are from Wilcoxon tests and Chi-2 or Fisher exact tests

#### Calibration

PP of the ES I and II is detailed in Table 5 for the overall population and according to age groups.

**Table 5:** Calibration of ES I and ES II for in-hospital mortality among the overall population and according to age.

	Overall population	<80	≥80
N =	7161	6329	832
**EuroSCORE I**			
O/E°	0.77 [0.77–0.85]	0.82 [0.73–0.91]	0.62 [0.49–0.78]
Hosmer-Lemeshow*			
p calibration ES I	<0.0001	0.001	<0.0001
**EuroSCORE II**			
O/E°	1.10 [0.99–1.21]	1.10 [0.98–1.22]	1.10 [0.87–1.37]
Hosmer-Lemeshow*			
p calibration ES II	0.006	0.13	<0.0001

°: Observed and Expected mortality ratio and [95% CI]

*: Hosmer-Lemeshow goodness-of-fit test

Values of O/E ratio and their 95% CI show a significant over-prediction of in-hospital mortality with the ES I in the overall population and in both age groups. This is consistent with the highly significant p values of the HL test. Calibration was better with the ES II, as shown by the values of O/E ratio and the fact that 95% CI included 1.0, although HL test was significant in the overall population and in patients aged over 80.

**Fig 2:**
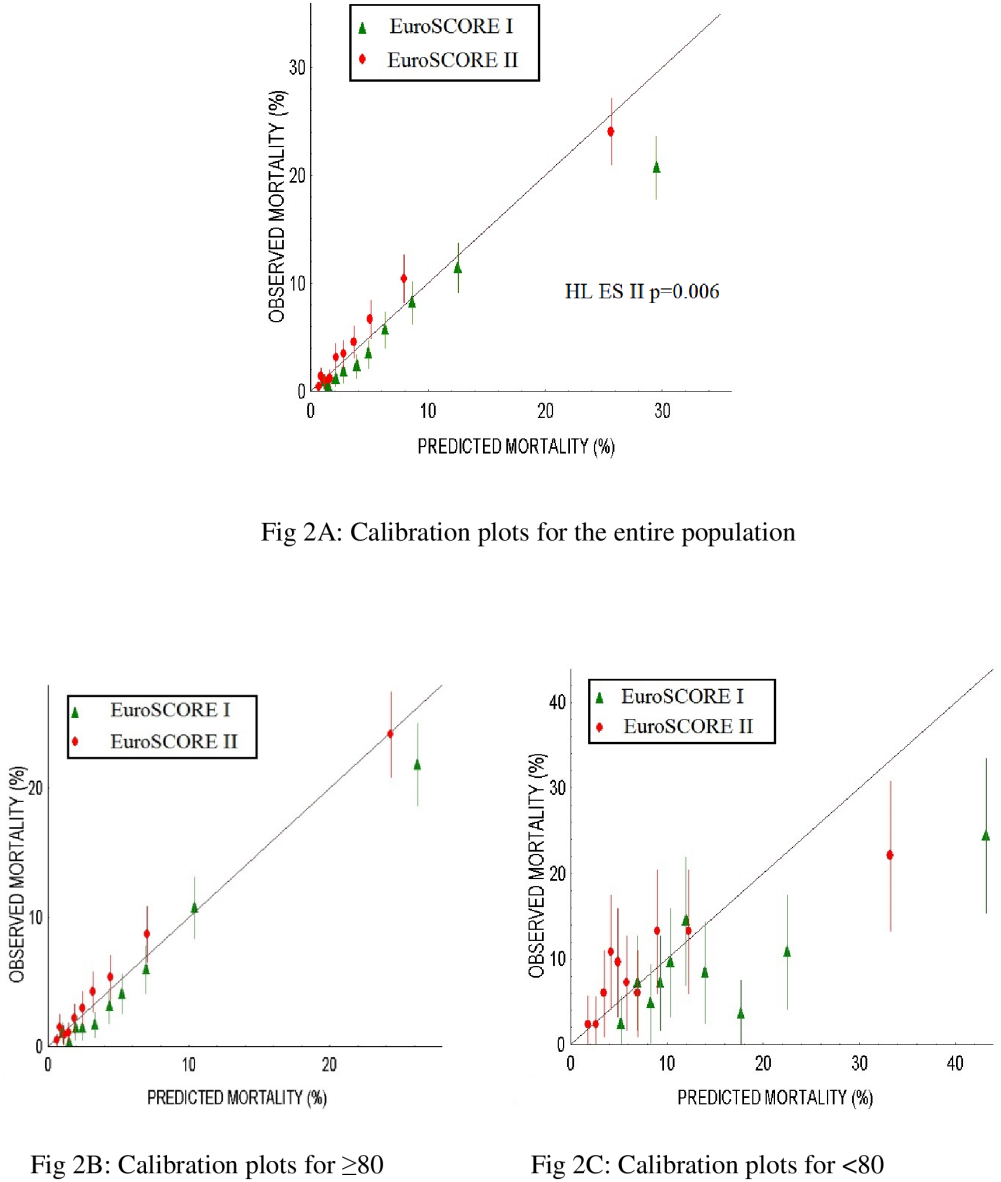
Calibration plots for the two models, for the entire population (Fig 2A) and the two studied groups (Fig 2B: ≥80 and Fig 2C: <80).

The calibration plots are represented for the whole population and for both age study groups in Fig 2.

The ES I shows a constant trend to over-prediction of in-hospital mortality, which is particularly marked in the high-risk group (predicted mortality >30%).

Conversely, the ES II had a better calibration than ES I. It had a tendency to under-estimate the in- hospital mortality but was otherwise close to the ideal diagonal except for the high-risk group.

The best predictive performance of ES II was observed in patients aged<80 with a more satisfying calibration among all risk deciles, showing a good agreement between observed and predicted in-hospital mortality, which is consistent with O/E values and the non-significant HL test.

In patients aged over 80, calibration was better with ES II than ES I as assessed by O/E ratios and 95% CI, although HL test was significant with both ES I and ES II. There was better agreement between observed and predicted mortality, as illustrated by CI of calibration plots (Fig 2) when considering patients with a predicted operative mortality of < 10% that represented 653 patients (78.4%) of those ≥ 80.

## Discussion

Cardiac surgery care providers are confronted with the constant increase of octogenarians in their daily practice, since such elderly patients represent almost 10% of their surgical procedures. Despite the increase in number of high-risk patients, the mortality rate has decreased, indicating an improvement in surgical, anesthetic and perioperative care.

Preoperative risk stratification of elderly patients is crucial because of medical, socio-economic and ethical considerations. Clearly, if the elderly are at higher risk of in-hospital complications and mortality [11], they must no longer be denied for surgery on the isolated age criteria, even in case of complex or emergent surgery [12–14]. If they leave the hospital alive, they could expect a longer life expectancy, a high discharge home rate after appropriate cardiac rehabilitation, and a good quality of life [15, 16].

Accurate risk prediction tools are essential because the elderly are more and more candidates for invasive procedures, except those with severe neurological or mental disorders or limited life expectancy. Credible therapeutic alternatives to conventional surgery such as percutaneous treatment of valvular heart disease are currently available. In the light of this recent evolution, accurate and objective information is mandatory for the patients and their families, guiding clinical decision making and, probably offering several choices to the intermediate-risk patients in a near future.

Obviously, a risk score should only be one of the components of the final decision. Europeans’ recent guidelines privilege the clinical judgment of multidisciplinary heart team for the evaluation of high risk patients [17]. Nevertheless, multifactorial risk models developed through multiple logistic regression analyses have been proposed to improve risk prediction, mainly the STS Risk Score and the ES II and they are widely used to provide an individual assessment of OM and therefore contribute to decision making for surgery.

The term of reference for the ideal risk score should be the simplicity of use (restricted numbers of parameters) and a robust PP.

The Society of Thoracic Surgeons (STS) has developed risk models to predict mortality and morbidity for CABG, valve, and combined valve and CABG operations from a large multicenter dataset of patients. The STS risk is regularly updated and used an on-line risk calculator including a large number of covariates. Even though it is rather time consuming to calculate it for each patient, the STS score seems to be a more reliable tool to assess the OM of the high risk patient than the ES I [18].

The ES II scoring system [8] was derived from a multicenter database of more than 20000 consecutive patients. It replaced the previous version elaborated 15 years earlier, for which poor calibration was demonstrated when it was applied to contemporary data sets. As recently reported [9, 19, 20], the ES II achieves good discrimination for OM but a lower calibration for high-risk surgery. Interestingly, Barili et al.[9] reported a high discriminatory power (0.82, 95% CI: 0.80–0.85) similar to the previous original version, and an optimal calibration up to 30%-predicted mortality.

Ours results are close to the discrimination values reported by Barili et al. with an AUC of 0.79 for the ES I and 0.80 for the ES II and 0.80 for the ES I and 0.81 for the ES II for the whole population and the patients aged<80 respectively.

At the age of 80 or more, we also report moderate discrimination of ES II with an AUC below 0.70 and a fair calibration (assessed with calibration plot) without improvement between ES I and ES II.

In a recent paper Poullis et al. [21] underlined an unacceptably low c-statistic index (<0.70) in patients aged ≥70 for both ES I and ES II but a good calibration regardless of age. For the first time, the authors choose not to sub- analyze by operation type but specifically by age, as we did.

However, we thought it would be better to focus on the elderly patients with a higher cutoff value for age (≥ 80 in our study as compared to ≥ 70 in their analysis). Although there are commonly used definitions of old age, there is no general consensus regarding the age at which a person becomes old.

Clearly, the first conclusion that could be drawn from our results and those of the literature is that the ES II has a poor PP among the octogenarians, as the others high-risk subjects [9, 20, 22].

What reasons could be put forward to explain such a failure of risk scoring among the elderly?

Experts explain this lack of performance by the fact that octogenarians are underrepresented in contemporary dataset with patients’ characteristics and operative techniques moving over time [17]. Moreover, specifics risks factors (extensive ascending aortic or valvular calcifications, tissue fragility) among the elderly have relevant impact on OM, but are not applicable to the general population. We could speculate that the high rate of patients (more than 25%) who died in the operating theater or very soon thereafter from direct surgical causes illustrate these unpredictable risks.

Finally, assessment of cognitive and functional capacity and indices of frailty are not evaluated by current risk scores. Recent works [23] have shown the negative impact on mortality of what is currently named the “frailty phenotype”. Preoperative evaluation with validated indices is mandated and is one of the components of the Heart Team specifications’[5].

Does that mean that all octogenarians (or more) need such a complex and, on a practical point of view, time consuming evaluation?

First of all, in order to assess all the calibration properties of the ES II system, we performed a graphical representation of O/E mortality by deciles of predicted probability (HL goodness-of-fit test, Fig 2) for the whole population and for the two age groups (<80 and ≥80). Using the same graphical representation, Barili et al. evidenced an optimal calibration until 30%-predicted mortality. Our results slightly differ from those of Barili in our specific elderly population: miscalibration of ES II is mainly observed when considering patients with a predicted OM of more than 10 percent (that is 20% of our population). In octogenarians, ES II provides a reliable estimation of OM, provided it is < 10%, which represents the vast majority of candidates to surgery.

To summarize clinical judgment and risk score calculation would be sufficient for the low- intermediary risk patients. Finally, further studies are needed to investigate if biological variables (such as hemoglobin level [24], serum albumin and high sensitivity C-reactive protein [25]) used as surrogate markers of frailty could add to the PP of the ES II.

### Limitations

The main limitations of our study are its retrospective and single-center nature. Furthermore, our results may not represent general practice or outcomes: we have to admit the dynamic nature of the outcome and its dependency on perioperative factors, which are modifiable in experts' hands. Our institutional in-hospital mortality rate was 5.7%, very close to the predicted mortality rate expected by the ES II (5.17%), higher than those of previous studies [8, 9, 22] but similar to that of a recent Spanish cohort [26], reflecting a much higher risk profile population.

This monocentric study insures the quality and the exhaustiveness of data collection particularly concerning the mortality rate. Our results differ from those previously reported in the ES II study: for the three study groups, we report a gap of 1% between 30 day mortality and in-hospital mortality for the whole population and, of 2% among the elderly, with in- hospital mortality being superior to the 30 days mortality. This raises question about the way in which the mortality was recorded for the ES II study, (the death in the hospital where the operation took place being the outcome measure) and the robustness of their observed mortality rate. A recent editorial underlines the crucial issue of a precise definition of such named OM among studies, for benchmarking between different cardiac surgical centers [27].

## Conclusion

The ES II has a better PP than the ES I in patients aged< 80. Its discrimination and calibration are less satisfying in patients aged≥80, showing an overestimation in the elderly at very high-surgical risk. Nevertheless, it shows an acceptable calibration until 10%- predicted mortality and could be used for the low-intermediate risk octogenarians, who represent the majority of patients in this age group. The poor discrimination and calibration of the ES II in very high-risk patients ≥ 80 should be an incentive to use the ES values with extreme caution when taking medical decision (e.g. TAVI versus conventional surgery) or informing the patients/families.

## Supporting information

S1 Table Dataset. Patient’s data.
(XLS)
